# Exchange Transfusion: A Good Option for the Acute Treatment of Familial Chylomicronemia Syndrome in the Neonatal Period

**DOI:** 10.7759/cureus.57019

**Published:** 2024-03-27

**Authors:** Sezai Arslan, Yasemin Abuşoğlu, Konca Altınkaynak, Hasan Kahveci

**Affiliations:** 1 Department of Inherited Metabolic Diseases, Erzurum City Hospital, Erzurum, TUR; 2 Department of Nutrition and Dietetics, Erzurum City Hospital, Erzurum, TUR; 3 Department of Medical Biochemistry, University of Health Sciences Erzurum Medical Faculty, Erzurum, TUR; 4 Department of Neonatology, University of Health Sciences Erzurum Medical Faculty, Erzurum, TUR

**Keywords:** lpl gene, hyperlipoproteinemia type 1, exchange tranfusion, lipoprotein lipase deficiency, familial chylomicronemia syndrome

## Abstract

Familial chylomicronemia syndrome (FCS) is one of the rare causes of hypertriglyceridemia. Plasmapheresis is recommended in patients with triglyceride levels greater than 2000 mg/dL. However, plasmapheresis is difficult to perform in most centers due to technical inadequacies in the neonatal period. There are some reports in the literature on the efficacy of exchange transfusion.

The index case involves a 20-day-old male patient who was admitted to the emergency department for restlessness and poor feeding. He was born at term with a birth weight of 4000 g. He was exclusively breastfed. The patient was taken to the neonatal intensive care unit due to his plasma being in the form of excessive lipemia. The first measurable triglyceride level was 5100 mg/dL (57.6 mmol/L). Breast milk was restricted, and intravenous hydration was started. However, his triglyceride level did not decrease despite this treatment. Other laboratory values could not be read due to excessive lipemic serum. On the third day of hospitalization, an exchange transfusion was decided upon in this case due to the development of respiratory distress (oxygen support, tachypnea). After exchange transfusion, the patient's triglyceride level reduced dramatically to 592 mg/dL (6.6 mmol/L), and his respiratory symptoms resolved.

The aim of this case report is to demonstrate that exchange transfusion therapy is a safe and effective treatment modality in the neonatal period for the acute management of FCS. Furthermore, dietary therapy restricted to long-chain fatty acids combined with medium-chain fatty acid supplementation is highly effective in the chronic management of these patients.

## Introduction

Familial chylomicronemia syndrome (FCS) is a rare disorder of lipoprotein metabolism characterized by triglyceride levels above 1000 mg/dL (11.2 mmol/L), especially in untreated patients. The estimated incidence is 1-2 per 1,000,000 [1-8]. It is inherited as an autosomal recessive trait. The most common mutations are in the LPL gene, which affects the synthesis of lipoprotein lipase. Less commonly, mutations in the APOC2, APOA5, GPIHBP1, and LMF1 genes have been associated with the disease [2-8]. Secondary hypertriglyceridemia can be caused by obesity, diabetes, hypothyroidism, Cushing's syndrome, nephrotic syndrome, and certain drugs (anabolic steroids, estrogen, L-asparaginase).

Lipid molecules absorbed from the gut after ingestion are transported through the portal system to the liver in the form of chylomicrons, with triglycerides comprising over 90% of the lipids in chylomicrons. Chylomicrons are cleared from the plasma after an overnight fast. The presence of chylomicrons in plasma after more than 12 hours of fasting is the main feature of chylomicronemia [1]. Other lipoproteins (VLDL, IDL, LDL) are responsible for transporting fatty acids and lipid molecules into cells.

Patients often present with restlessness, vomiting, and milky-looking plasma after breastfeeding or formula feeding in FCS. Eruptive xanthomas, lipemia retinalis, hepatosplenomegaly, gastrointestinal hemorrhage, and seizures may be seen [6-10]. Recurrent attacks of pancreatitis are among the most dangerous complications. Hypertriglyceridemia is thought to increase pancreatic lipase enzyme activity, causing inflammation in pancreatic capillary and acinar cells due to increased viscosity. The mortality rate due to acute and chronic pancreatitis has been reported to be as high as 6%. Diet restricted in long-chain fatty acids (8-15% of daily energy requirement from lipids) with medium-chain triglyceride (MCT) supplementation is effective in the chronic management of these patients.

Acute management of familial chylomicronemia is important. Plasmapheresis is recommended for patients with triglyceride levels in excess of 2000 mg/dL [3]. However, plasmapheresis cannot be performed in many centers due to hemodynamic instability and technical inadequacies in the neonatal period. Publications report the efficacy of exchange transfusion (ET) treatment, with ET reported to have fewer side effects than plasmapheresis [4-10]. In this case report, we emphasize the efficacy of exchange transfusion in the acute treatment of familial hyperchylomicronemia in the neonatal period.

## Case presentation

**Figure 1:**
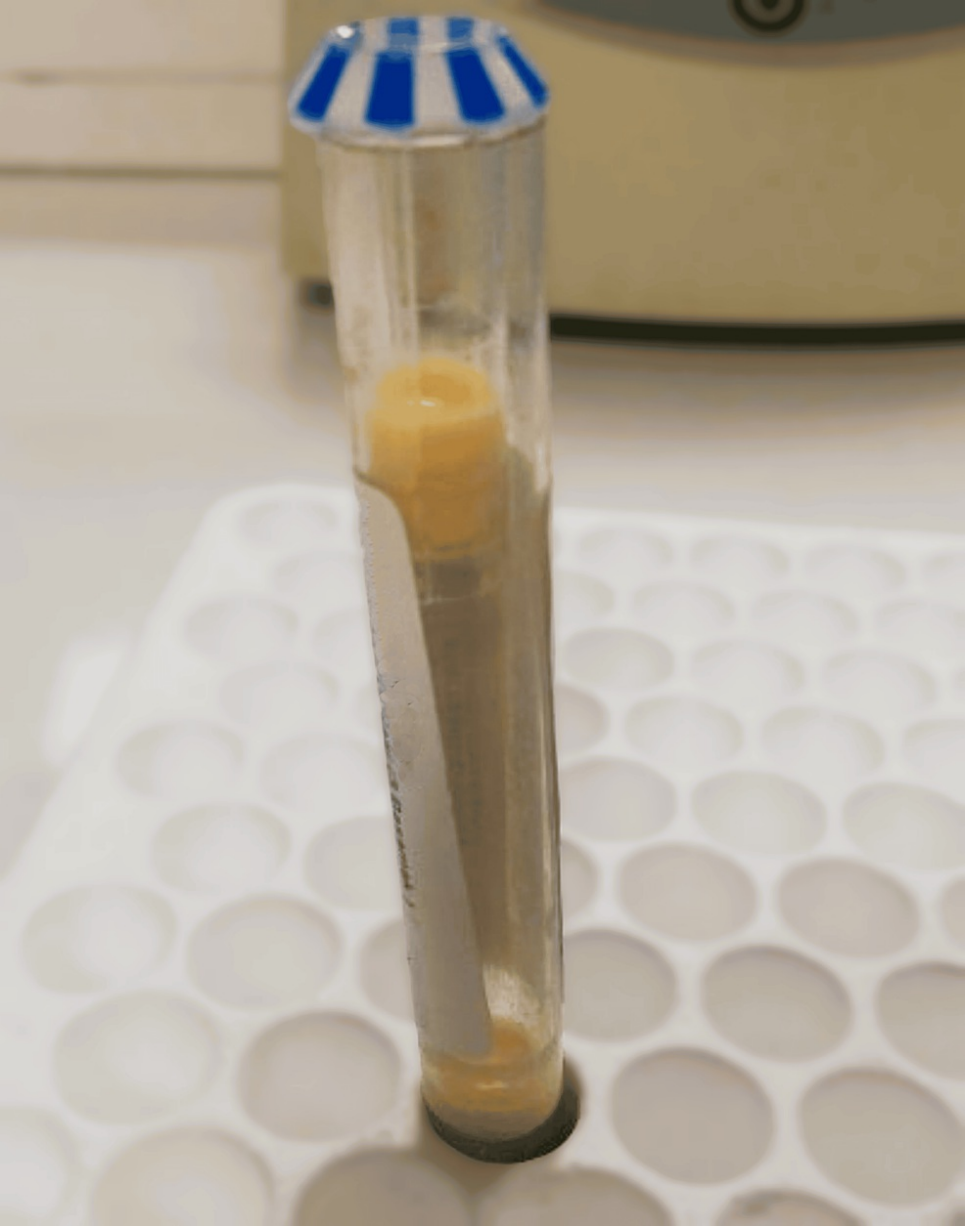
Milky-creamy appearance of the case's plasma

A 20-day-old male patient was admitted to the emergency department for poor appetite and restlessness. He was exclusively breastfed. There was no fever, vomiting, or diarrhea. The patient was started on intravenous hydration and routine blood tests were performed. Venous blood gas showed the following results: pH: 7.41, PCO_2_: 40.7 mmHg, HCO_3_: 25.3 mmol/L, lactate: 2.2 mmol/L, and capillary glucose: 62 mg/dL. Other laboratory tests could not be evaluated because the patient's serum was extremely lipemic (Figure 1). The patient was admitted to the neonatal unit for careful monitoring.

The patient was born at term with a weight of 4000 g. There were no prenatal and postnatal problems. The parents were consanguineous, and he has three healthy siblings. The triglyceride level of the patient's father was 900 mg/dL. Triglyceride levels were in the normal range for his mother and siblings.

**Figure 2:**
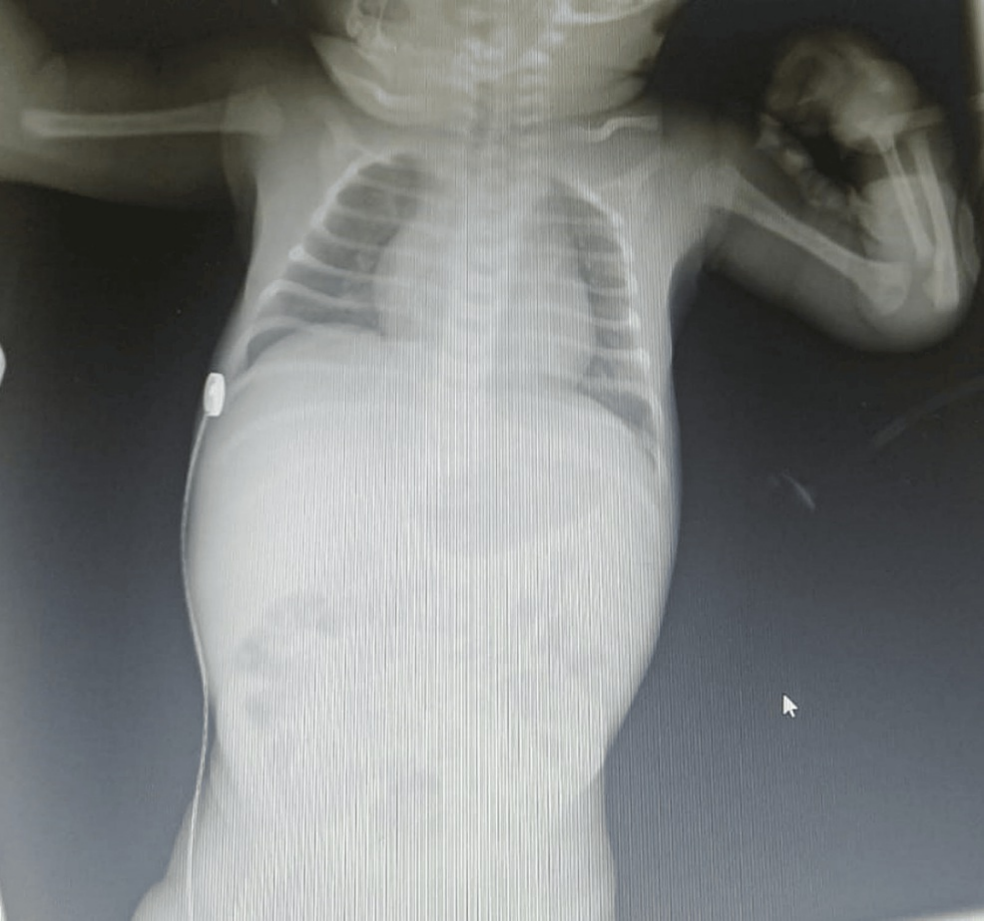
X-ray image of the patient's chest

During the patient's follow-up, the triglyceride level remained greater than 550 mg/dL. The first triglyceride level measured by the dilution method was 5100 mg/dL (57.6 mmol/L). His other measured lipid levels were total cholesterol 595 mg/dL, and HDL 71 mg/dL. Antibiotic treatment was started when CRP was 54.6 mg/dL (0-1). The abdominal ultrasound showed grade 1 pelviectasis of the left kidney with a minimal increase in liver echogenicity. His chest radiography was normal (Figure 2). Echocardiography showed a secundum ASD (atrial septal defect) of 4 mm diameter. His triglyceride level was 4710 mg/dL (53.2 mmol/L) despite breast milk restriction and intravenous hydration therapy. On the third day of hospital admission, an exchange transfusion was decided upon due to deterioration in the general condition, cyanosis, and respiratory distress.

ET is a procedure that replaces the baby's blood with donor blood by repeatedly withdrawing and exchanging small amounts of blood over a short period of time. We used A Rh(+) packed erythrocytes collected five days before the procedure, filtered, irradiated, and suspended in A Rh(+) plasma at a rate to achieve a hematocrit of 40%. Up to 5 mL/kg of blood was exchanged with infant blood each time, for a cumulative exchange volume of 2x80 mL/kg, i.e., twice the estimated infant blood volume. ET was performed percutaneously under ultrasound guidance without vein ligation using a double-lumen 5 French central venous catheter inserted into the right internal jugular vein. A pull-push technique was used with a special four-way valve. The procedure took 120 minutes. There were no complications after the ET procedure.

**Figure 3:**
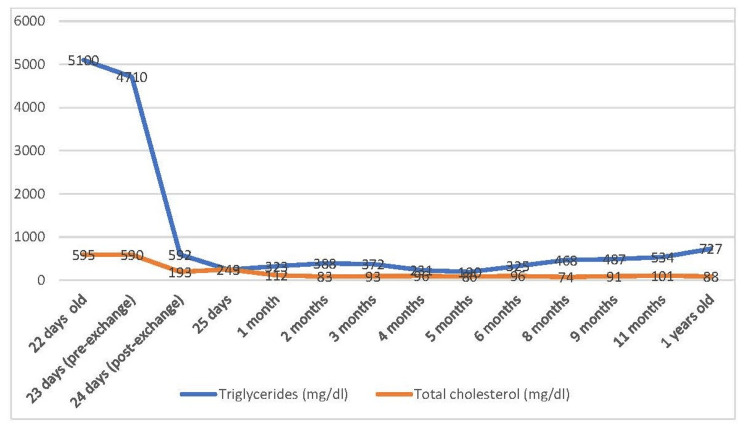
Triglyceride and total cholesterol levels of the case before and after exchange transfusion

After the exchange transfusion, the patient's triglyceride level dropped dramatically to 592 mg/dL (6.6 mmol/L) (Figure 3). The general condition improved, and the respiratory distress disappeared. Other blood parameters could be read by laboratory equipment (Table 1). Breast milk was severely restricted in the patient's diet, and special formulas (lipid-free and maltodextrin) were used to provide 8-10% of total energy from lipids. Walnut oil was used to provide the essential fatty acid requirement. MCT oil was added to include 50% of the total lipid content. Multivitamin support including fat-soluble vitamins was started.

**Table 1:** Other blood and biochemistry results (after exchange) of the case WBC, white blood cells; PLT, platelets; ALT, alanine aminotransferase; AST, aspartate aminotransferase; CRP, C-reactive protein; PT, prothrombin time; aPTT, activated partial thromboplastin time; INR, international normalized ratio.

Test	Observed value	Normal range
Hemoglobin, g/dL	15	14.1-17.8
Hematocrit, %	39.5	36.4-47.2
WBC, 10^9^/L	5650	4390-11,590
PLT, 10^9^/L	101	152-383
Glucose, mg/dL	72	70-106
Urea, mg/dL	5	4-18
Creatinine, mg/dL	0.16	0.7-1.3
ALT, U/L	18	(10-49)
AST, U/L	30	(25-90)
Total, mg/dL	1.97	0.2-6.1
Direct bilirubin, mg/dL	0.53	0-0.3
Na, mmol/L	145	136-145
K,mmol/L	3.5	3.5-5.1
Amylase, U/L	<20	4.4-55
Lipase, U/L	19	12-53
CRP, mg/L	16	0-1
Vitamin A, µg/dL (at 3 months of age)	29	(>20 in familial chylomicronemia) [13]
Vitamin E, mg/dL (at 3 months of age)	0.98	(>0.6 in familial chylomicronemia) [13]
Vitamin D, ng/mL (at 3 months of age)	38.2	30-60
PT, seconds (at 7.5 months of age)	133.8 s when vitamin K was administered (control PT: 26 s)	24-36
aPTT, seconds (at 7.5 months of age)	14.2	12-16
INR	1.06	0.8-1.2

**Figure 4:**
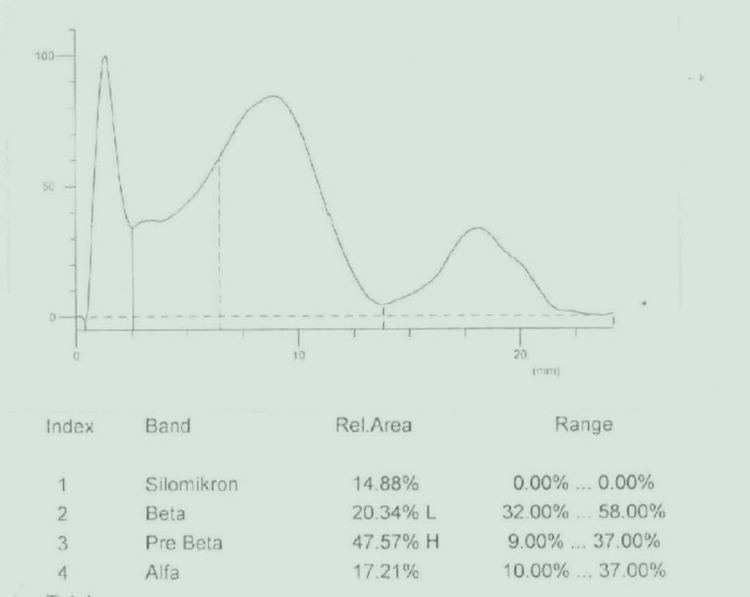
The result of lipid electrophoresis of the patient

**Figure 5:**
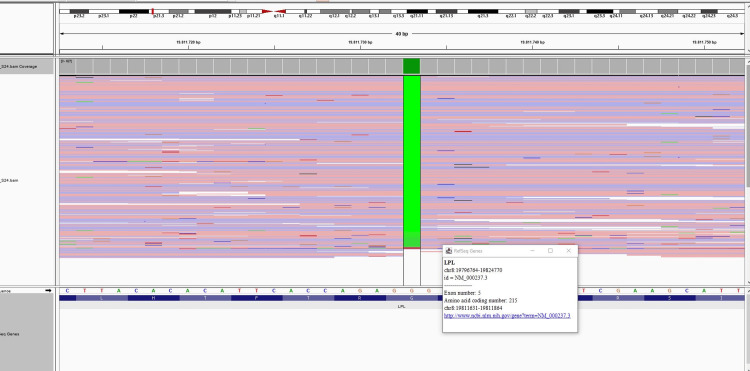
The sequencing chromatograms of the mutation in the patient

The patient's lipoprotein electrophoresis showed an increase in chylomicrons of 14.88%. Beta-lipoprotein was 20.34% (32-58), pre-beta-lipoprotein 47.57% (9-37), and alpha-lipoprotein 17.21% (10-37); this result is consistent with the type 1 hyperlipoproteinemia pattern (Figure 4). Genetic testing was performed for the etiology of hypertriglyceridemia. DNA was isolated from EDTA blood samples by the magnetic bead method (MagPurix- Zinexts, Taiwan). “PRIMER© - Primer Designer v.2.0 (Scientific & Educational Software)” software was used for primer design. Primers were designed, optimized, and used in routine studies. In this study, the MiSeq (Illumina, San Diego, CA) system was used, and the targeted resequencing method was applied. An in-house primer set was used for target amplification. Sequence differences are evaluated as disease-causing and non-disease-causing changes during reporting. ACMG criteria are used to evaluate whether these changes are the cause of the disease. Genetic analysis revealed a homozygous c.644G>A (p.G215E) pathogenic mutation in the LPL gene, and this mutation was evaluated as pathogenic according to ACMG criteria. Both parents were heterozygous carriers of the p.G215E mutation (Figure 5). The diagnosis was confirmed as familial chylomicronemia due to lipoprotein lipase deficiency.

His neuromotor development was compatible with his peers at the last visit. Growth parameters were within the normal range at 11 months of age (height: 75 cm +0.05 SD, weight: 8.5 kg -1.08 SD). The increase in echogenicity in the patient's liver had disappeared in the abdominal ultrasound performed later. Lipemia retinalis was not observed in repeated eye examinations.

## Discussion

Lipids are one of the body's main building blocks. Lipids include cholesterol, cholesterol esters, triglycerides, and phospholipids in plasma. However, lipids are insoluble in water, so they are transported as larger, complex, water-soluble molecules called lipoproteins in the plasma. These lipoproteins (VLDL, LDL, IDL, HDL) have different densities due to their varied protein and lipid contents. Fatty acids, absorbed from the intestine, enter the bloodstream as chylomicrons via the lymphatic system. Most chylomicrons are removed from circulation by lipoprotein lipase, found in the heart, muscle, and adipose tissue. The remaining chylomicron remnants are transported to the liver by lipoproteins [7].

Patients with familial hypertriglyceridemia may present with growth retardation, abdominal pain, vomiting, hepatosplenomegaly, eruptive xanthomas, and lipemia retinalis in early childhood. A patient, aged seven weeks, with grade three lipemia retinalis and a mutation similar to our patient's, has been reported in the literature [5]. However, fundus examination was normal in our patient. Lipemia retinalis did not develop in subsequent examinations. We thought this might be related to the fact that our patient presented at three weeks of age or sooner.

Mutations in the LPL gene are found in 80% of patients with familial hypertriglyceridemia. More than 220 pathogenic variants (missense, nonsense, splice variants, deletions, insertions, duplications) associated with the LPL gene have been identified. Our case has a homozygous pathogenic mutation of the LPL gene, c.644G>A (p.G215E), previously described in the literature.

Severe hypertriglyceridemia has been reported to lead to decreased lung function, hemolysis, liver dysfunction, and an increased risk of sepsis in neonates. Respiratory symptoms are thought to be related to decreased diffusion capacity as a result of fat emboli in the pulmonary capillaries. Additionally, a decrease in nitric oxide bioavailability, leading to vascular relaxation, and an increase in inflammatory mediators (prostaglandins, thromboxane) [10,11] are also important. In our case, tachypnea, decreased oxygen saturation, and cyanosis were observed on day three of hospitalization. Respiratory findings improved after exchange transfusion.

Hypertriglyceridemia (>1,000 mg/dL) can lead to life-threatening attacks of pancreatitis. Emergency treatment is important for extremely high triglyceride levels (>2,000 mg/dL). Plasmapheresis is recommended for acute treatment, but it cannot always be used because the hemodynamic structure of neonates does not allow for large blood withdrawals, and the plasmapheresis machine is not compatible with the patient's weight [2-12]. Our patient required urgent treatment due to deterioration in general condition, respiratory distress, and decreased oxygen saturation. Plasmapheresis was not available in our unit. Since exchange transfusion has been reported in the literature [4-6] to be effective in the acute treatment of hypertriglyceridemia and is less invasive than plasmapheresis, exchange transfusion was performed on our patient. In agreement with the literature, the patient's triglyceride level decreased dramatically after exchange transfusion, and hemogram and other laboratory tests could be read on the machines.

Exchange transfusion has been widely used by neonatologists to treat indirect hyperbilirubinemia in newborns since 1940. ET has significantly prevented mortality due to hemolytic anemia and complications of kernicterus. The most common side effects reported with ET are thrombocytopenia, hypocalcemia, hyperkalemia, apnea, bradycardia, hypotension, and catheter-related complications. In our case, mild thrombocytopenia was observed after the procedure and resolved spontaneously.

His diet was adjusted to foods with low lipid content (Milupa® Basic-F, Nutricia® Fantomalt, Nutricia® Protifar) during the chronic follow-up of our patient. Total daily energy was organized as 65-70% from carbohydrates, 15-20% from protein, and 8-15% from lipids. Essential fatty acid requirements were supplied by walnut oil, which is rich in linoleic acid (LA) and alpha-linolenic acid (ALA) and provides 3-4% of total energy [13]. Essential fatty acid deficiency is associated with inadequate growth, dry/dull hair, dry/scaly skin, soft brittle nails, and impaired wound healing. Our case was given walnut oil from 1 month of age, although it is given from 8 months of age in similar cases in the literature [14]. No intolerance to walnut oil was observed.

Dietary management of patients with familial lipoprotein lipase deficiency restricts long-chain fatty acids and provides medium-chain fatty acids. MCT oil contains caprylic acid (C8) and capric acid (C10). Medium-chain fatty acids are oxidized to ketones, which enter the liver directly from the portal system without entering the chylomicron structure. The total energy requirement can be adjusted by the macro content of the diet without raising triglyceride levels when MCT oil is used in hypertriglyceridemia [15]. In our case, 50-60% of the total lipid was provided by MCT oil, and triglyceride levels were kept under control.

## Conclusions

Acute management of familial chylomicronemia is important. Plasmapheresis is recommended for patients with triglyceride levels in excess of 2000 mg/dL. However, plasmapheresis cannot be performed in many centers due to hemodynamic instability and technical inadequacies in the neonatal period. We would like to emphasize the efficacy and safety of exchange transfusion therapy in the acute treatment of hypertriglyceridemia in the neonatal period. Furthermore, a long-chain fatty acid-restricted diet combined with MCT supplementation to meet essential fatty acid requirements appears to control triglyceride levels in the chronic management of FCS.
